# Notes and News

**Published:** 1894-12-01

**Authors:** 


### Notes and News.

Collected and Communicated.

A new ward and dispensary was opened on the 16th ult. by Lord Derby at the Liverpool Hospital for Women. The institution is much resorted to, and the management economically conducted.

The new Infectious Diseases Hospital for the Rural and Urban sanitary authorities of Stratford-on-Avon has just been opened. The buildings, costing between £5,000 and £6,000, consist of five blocks providing accommodation for twenty beds.

Quite a novel privilege is to be introduced for the use of the inmates of the Dewsbury Infirmary. The Vicar and Churchwardens of St. Mark's Church have offered as an experiment to institute telephonic communication between the church and the infirmary, whereby 20 or more inmates will be able to listen to the services. The experiment is a most novel departure, and we shall look with interest to the result.

Sir Walter Foster, M.P., was usefully engaged on Saturday, when he visited Sheffield to inaugurate a new scheme of the guardians for dealing with pauper children. The ideal system, no doubt, of dealing with such children is boarding out in country districts, but many town guardians, not entirely without reason, hesitate to send their charges so entirely out of the reach of supervision by themselves or their own officers, and the Sheffield guardians have endeavoured to solve the difficulty by taking cottages in various parts of the city, in the midst of the artizan population, where children who come under their care will be sent without entering the workhouse. In these homes they will be attended by foster parents, sent to public elementary schools and places of worship, and in every way brought up like the children of the working classes. We hope they will be taught a trade. One good side of the country boarding-out is that the children learn the trade of the district, agriculture, or whatever it may be. Even the Grimsby apprenticeship system, bad as it is in many respects, teaches a way of earning a living. Unless education ends by a trade it is of small avail.

At the eighty-fifth anniversary of the Derbyshire Royal Infirmary, a proposition to erect a chapel for the Infirmary to the memory of the late Sir William Evans, was adopted. It is to cost £2,000. In the completed portion of the new Infirmary 120 beds are now available.

Patent medicines are treated with severity in the United States. In Iowa a Bill has just been passed, requiring them to have a printed statement affixed giving the ingredients of their preparation. The penalty is a fine, limited to £20, or imprisonment for six months. If such regulations were enforced in England, such cases as the one recently reported of the child who fell a victim to a soothing syrup containing opium, would be of less frequent occurrence.

At the Council meeting of the Metropolitan Sunday Fund on Monday, it was decided to hold the next Hospital Sunday on June 16th, 1895. All the members were re-elected at the meeting, the Rev. C. H. Grundy, of St. Peter's, Brockley, taking the place of the late Canon Rowsell. A letter of protest from Lord Knutsford, against a grant to the Queen's Jubilee Hospital, was referred to the Distribution Committee. The meeting was well attended, though some prominent members were unavoidably absent.

At a special meeting of governors of the Richmond Royal Hospital, at which the Duke of Teck presided, it was decided to commence the additional buildings, consisting principally of domestic and administrative accommodation required to make the institution complete. The money has not yet been subscribed, and to obtain the additional sum needed. a loan from the bank has been arranged, at very moderate interest. It remains for some generous friend to come forward and clear the hospital from this liability.

The proceedings at the special meeting of governors of the Salisbury Infirmary "to consider the election of a chaplain," as reported in the local press, have been justly criticised and condemned. It appears that, in response to an advertisement, fifty-nine candidates presented themselves for the appointment. Despite a protest by Canon Bernard, Mr. Manning, and other governors, who wished to refer these fifty-nine applications to a committee to go carefully into them, to examine all the testimonials and to report, the governors present determined that it was a waste of time to read any testimonials, and that the election should proceed forthwith. This resolution was come to, although the chairman stated that the testimonials had not even been read by the committee or by anyone. One gentleman was then proposed, seconded, and elected to the office of chaplain without his testimonials being read or the governors having any knowledge of the fifty-eight other candidates, their names, claims, and qualifications. These proceedings were probably the most irregular that have ever taken place at any court of governors of any Institution in this country. We feel that they cast a most grievous reflection upon the majority of those who took part in them, and that the character for fairness and business capacity of the whole body of governors of the Salisbury Infirmary is at stake. Will not some independent governor move for a small committee on bye-laws, that regulations may be enacted to prevent any repetition of such scandalous proceedings in the future?

